# Detection of MicroRNAs in Archival Cytology Urine Smears

**DOI:** 10.1371/journal.pone.0057490

**Published:** 2013-02-28

**Authors:** Francesca Simonato, Laura Ventura, Nicola Sartori, Rocco Cappellesso, Matteo Fassan, Lill-Tove Busund, Ambrogio Fassina

**Affiliations:** 1 Department of Medicine (DIMED), Surgical Pathology & Cytopathology Unit, University of Padua, Padua, Italy; 2 Department of Statistics, University of Padua, Padua, Italy; 3 Department of Pathology & Diagnostics, Surgical Pathology & Cytopathology Unit, University of Verona, Verona, Italy; 4 Department of Clinical Pathology, University Hospital of North Norway, Institute of Medical Biology, University of Tromso, Tromso, Norway; University of Verona, Italy

## Abstract

MicroRNAs’ dysregulation and profiling have been demonstrated to be clinically relevant in urothelial carcinoma (UC). Urine cytology is commonly used as the mainstay non-invasive test for secondary prevention and follow-up of UC patients. Ancillary tools are needed to support cytopathologists in the diagnosis of low-grade UC. The feasibility and reliability of microRNAs profiling by qRT-PCR analysis (miR-145 and miR-205) in archival routine urine cytology smears (affected by fixation/staining [Papanicolau] and room temperature storage) was tested in a series of 15 non-neoplastic and 10 UC urine specimens. Only samples with >5,000 urothelial cells and with <50% of inflammatory cells/red blood cells clusters were considered. Overall, a satisfactory amount of total RNA was obtained from all the considered samples (mean 1.27±1.43 µg, range 0.06–4.60 µg). Twenty nanograms of total RNA have been calculated to be the minimal total RNA concentration for reliable and reproducible miRNAs expression profiling analysis of archival cytological smears (slope = -3.4084; R-squared = 0.99; efficiency = 1.94). miR-145 and miR-205 were significantly downregulated in UC samples in comparison to non-tumor controls. These findings demonstrate that urine archival cytology smears are suitable for obtaining high-quality RNA to be used in microRNAs expression profiling. Further studies should investigate if miRNAs profiling can be successfully translated into clinical practice as diagnostic or prognostic markers.

## Introduction

Bladder cancer represents the fourth male most common cancer in the western world and urothelial cell carcinoma (UC; previously designated as transitional carcinoma [TCC]) accounts for approximately 95% of bladder malignancies and is the most common tumor detected by urine cytology [1]. Two distinct phenotypic and molecular pathways have been demonstrated in UC: i) the superficial papillary carcinoma, accounting for more than 80% of bladder tumors, with tendency to recur locally (approximately 70%), with rare invasion and metastasis; ii) and the invasive non-papillary bladder tumor, without known papillary precursor, which is commonly associated with carcinoma *in situ* (CIS) with unfavourable prognosis [2], [3].

Urine cytology is widely and routinely employed as: i) the preliminary analysis in the evaluation of patients presenting with haematuria or painful urination suggestive for urinary system pathology [4]; ii) screening test for the early detection of bladder cancer in selected populations exposed to known bladder carcinogens; and iii) the mainstay in the follow-up of patients with a history of malignancy involving the urinary tract [4], [5].

While the reported sensitivity and specificity of urine cytology in the diagnosis of high-grade UC exceed 90% [6], its sensitivity and specificity drastically drop-down for low-grade cases [7].

To date, several biomarkers, such as bladder tumor antigen (BTA) [8], nuclear mitotic apparatus protein 22 (NMP22) [9], ImmunoCyt™, and UroVysion™ [10], have been proposed to ancillary support the cytological diagnosis (in particular in the presence of low-grade lesions); however, none of these provided substantial help to the diagnostic process [3].

MicroRNAs (miRNAs) are a class of short (∼20 nucleotides), regulatory, non-coding RNA, involved in several cellular processes in both development and pathology [11]–[13]. Unlike most RNAs, miRNAs are long-lasting *in vivo* and very stable *in vitro*, which is critical in the clinical setting [14]. Indeed, miRNAs have been detected in whatever human specimen (tissue, blood, plasma, serum, sputum, feces, CSF, and urine) yielding their recognition relatively simple and often non-invasive [15]–[18]. Moreover, miRNA profiling methods have been showed to be standardizable and of clinical impact in human cancers [19], [20]. Altogether, such characteristics pinpointed miRNAs as suitable biomarkers to be detected even in scant degraded cytology samples affected by fixation/staining and room temperature storage such as routine diagnostics urine smears.

Since the pioneering report by Gottardo *et al.* [12], describing up-regulation of a set of miRNAs in 27 UC *vs* 2 normal bladder mucosa samples, to date, several large profiling studies in tumor resections and cell cultures have been described [21]–[26]. Among the others, the down-regulation of miR-145 has been demonstrated to reliable differentiate low-grade UC from normal urothelium [22], [25]. Instead, miR-205 has been reported to be early down-regulated during UC carcinogenesis [23], [24], but over-expressed in progressed tumors [12], [26].

In a series of non-neoplastic and UC urine archival cytological specimens, we tested the feasibility and reliability of profiling analysis of two down-regulated UC miRNA biomarkers (miR-145 and miR-205). Our data provide further evidence on miRNAs as a promising class of diagnostic, prognostic, and predictive biomarkers.

## Materials and Methods

### Ethics Statement

Written informed consent was obtained from all participants involved in the study, which was approved (number approval N 0002539) by the institutional review board of the University Hospital of Padua (Azienda Ospedaliera di Padova – Università degli Studi di Padova).

### Patients & Specimens

From the archives of the Surgical Pathology and Cytopathology Unit at Padua University (Cytopathology Unit) was retrieved a total of 10 consecutive low-grade UC and 15 consecutive non-neoplastic cytology samples reported in 2009 (>36 months old). Original routine cytological slides (Cytofix-fixed [Bio-Fix 05-x200, Bio-Optica, Milano, Italy] and Papanicolaou-stained) were jointly re-assessed by two pathologists (RC and MF) accordingly to the current WHO criteria [2], [27]; in cases where their opinions differed, a third expert cytologist (AF) was involved. All low-grade UC cytological diagnoses were also histologically confirmed in bladder biopsies sampled during the following cystoscopy [2].

**Figure 1:**
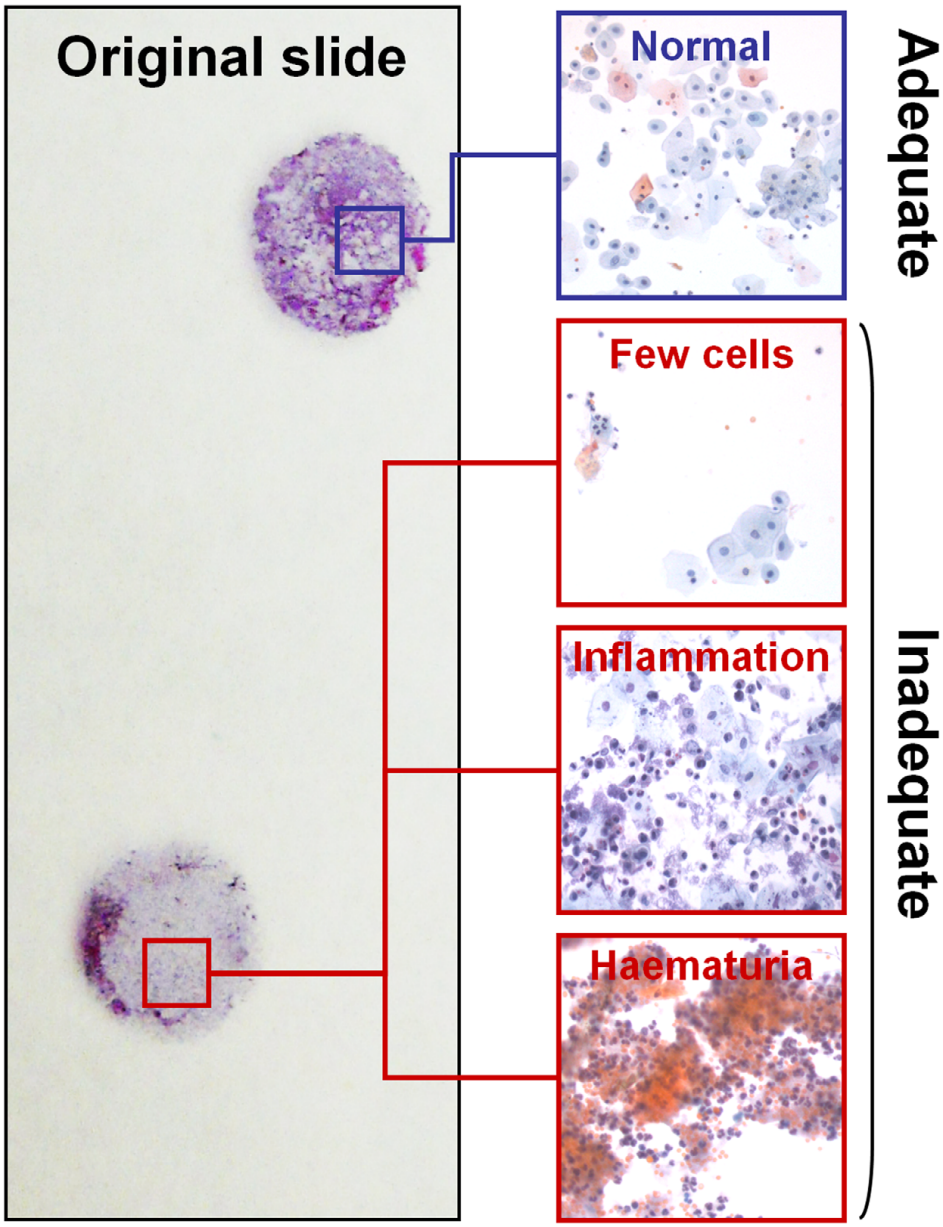
Selection of the cytology specimens. Only cases with >5,000 well-preserved and well-visualized urothelial cells (as observed in 10 fields at 4x magnification) were selected. Cases largely constituted by red blood cells or inflammatory cells were excluded.

The presence of >5,000 well-preserved and well-visualized urothelial cells (as observed in 10 fields at 4x magnification) were appointed as minimum accepted cellularity. In neoplastic specimens, only slides with a proportion of tumor cells >25% were selected. Slides with >50% of urothelial cells obscured by either white or red blood cells were excluded (Figure 1).

Overall, the male:female ratio was 8∶2 and mean age was 81.7±7.7 years (median = 84; range = 67–92) for UC patients, while male:female ratio was 8∶7 and mean age was 64.5±11.2 years (median 66; range 44–81) for the non-neoplastic patients.

### Cell Cultures

As reference for the qRT-PCR analyses, Capan-1 and BxPc3 cell lines were purchased from the American Type Culture Collection (ATCC, Manassas, VA, USA) and maintained as recommended. Cell proliferation and counting was determined by monitoring each cell line with the xCELLigence system (Roche, Mannheim, Germany). xCELLigence cell index impedance measurements were performed according to the instructions of the supplier. Cells (5×10^3^ cells/well) were seeded in 150 µl of the complete medium in a 16-well microtiter plate specifically designed to measure cellular impedance (E-plate; Roche) and placed into the xCELLigence system for data collection after a 30 min incubation at room temperature. The xCELLigence software was set to collect impendence data (reported as cell index) at least once every 15 min for 4 days. Overall, 10^6^ cells at exponentially growth phase was yielded from each cell line for total RNA extraction, after routine spotting, fixation, and staining (as for the urine samples).

### RNA Extraction

Coverslips were removed by immersion in xylene for 72 hours and the slides were rinsed three times in 95% ethanol. Cells on the slides were scraped in 1.5 ml tubes by using sterile razors and suspended in 500 µl of TRIzol reagent (Invitrogen, Carlsbad, CA) for total RNA isolation according to the manufacturer’s instructions [28]. Amount and purity of RNA was assessed by UV spectrometry (Nanodrop ND1000, Thermo Scientific, Wilmington, DE).

### Quantitative Reverse-transcription-PCR

RNA dilutions were retro-transcribed by using the VILO cDNA Synthesis kit (Invitrogen). To detect and quantify mature *hsa-miR-145* (miR-145; primer sequence: 5′- CAG TTT TCC CAG GAA TCC CT-3′) and *hsa-miR-205* (miR-205; primer sequence: 5′- CTT CAT TCC ACC GGA GTC TG -3′), the NCodeTM miRNA qRT–PCR method (Invitrogen) was used on the LightCycler 480 Real-Time PCR System (Roche Diagnostic, Mannheim, Germany), as reported elsewhere [19], [28]. The results were normalized with the small nuclear RNA U6B (RNU6B; primer sequence: 5′- ACG CAA ATT CGT GAA GCG TT -3′; Invitrogen). All the reactions were run in triplicate, including no-template controls. A total of seven serial RNA dilutions for each non-neoplastic urine sample (50 ng, 40 ng, 30 ng, 20 ng, 10 ng, 1 ng, and 0.1 ng) and a total of eight RNA dilutions for cell lines (50 ng, 40 ng, 30 ng, 20 ng, 10 ng, 5 ng, 1 ng, 0.1 ng) were tested for miR-205 and RNU6B in order to define the minimal adequate RNA quantity to use for miRNAs qRT-PCR analysis. To support the data, differences in miR-145 and miR-205 expression were tested in non-neoplastic and UC urine cytological specimens.

### Statistical Analysis

Statistical analyses for testing the qRT-PCR efficiency were based on the results of experiments on routine cytology samples and two cell lines. The experiments were performed separately for RNU6B, miR-145and miR-205. The threshold cycle (Ct) was plotted against the log10 amount of cDNA input (ldose). The focused parameter was the slope of the relationship between ldose and Ct, since it was used to estimate the efficiency *e* of the qRT-PCR, according to the equation *e* = –10/slope. The ideal slope is −3.32, which corresponds to an amplification efficiency of 100% (*e* = 2). A slope comprised in the range of −3.6 to −3.1 is considered acceptable for qRT-PCR results [29]. For the two cell lines we investigated the relationship between Ct and ldose through linear regression models. For the urine samples, linear mixed effects (LME) regression models including a random effect for the subject were used. This class of models is needed to deal with intra-individual variability. The best doses were selected in terms of the estimated slopes. A likelihood ratio test R2 was computed for LME to summarize how well the model explains the data [30]. The significance level for all statistical analyses was set at *α* = 0.05. Differential expression between non-neoplastic and UC samples were tested for both miR-205 and miR-145 using the Welsh two-sided t-test. All statistical analyses were performed using the R software (R Development Core Team, version 2.9; R Foundation for Statistical Computing, Vienna, Austria; www.R-project.org).

## Results

### Total RNA Obtained from Archival Urine Cytology Smears

As showed in Table 1, total RNA was successfully extracted from all the 25 urine cytological specimens and the mean RNA yield per sample was 1.27±1.43 µg (median = 0.88; range = 0.06–4.60), with a OD_260/280_ ratio of 1.96±0.34 (median = 1.94; range = 1.7–2.2). Only one sample presented a lower RNA purity with a OD_260/280_ ratio of 2.80 (case #9), but was retained in the analysis. As expected, the purity and quantity of extracted RNA were higher in the two cell lines (RNA obtained from 10^6^ cells, routinely fixed/stained and room temperature stored): CAPAN-1 RNA yield 10.8 µg and OD_260/280_ ratio of 1.85; BxPC3 RNA yield 15.2 µg and OD_260/280_ ratio of 1.92.

**Table 1:** Cytological and molecular features of the 25 cytology samples.

Sample	Type	RNA yield (µg)	A260/A280 ratio	Cellularity	Cell types	
					Cancer	Inflammatory
1	NT	1.28	1.74	H	–	5%
2	NT	1.52	1.80	H	–	10%
3	NT	0.88	1.01	L	–	–
4	NT	2.60	1.96	H	–	–
5	NT	2.92	2.30	H	–	5%
6	NT	4.60	2.24	H	–	30%
7	NT	4.44	2.25	H	–	20%
8	NT	0.08	1.90	L	–	10%
9	NT	0.29	2.80	L	–	–
10	NT	0.06	1.92	L	–	–
11	NT	2.21	2.01	H	–	–
12	NT	1.04	1.71	H	–	15%
13	NT	1.32	1.94	H	–	10%
14	NT	1.15	1.71	H	–	5%
15	NT	0.94	1.74	L	–	5%
16	T	0.17	2.22	L	40%	10%
17	T	0.11	2.25	L	30%	20%
18	T	0.25	2.25	L	50%	5%
19	T	0.16	2.00	L	35%	15%
20	T	0.13	2.24	L	70%	10%
21	T	0.25	2.01	L	60%	10%
22	T	4.20	1.88	H	50%	5%
23	T	0.16	1.68	L	30%	15%
24	T	0.48	1.66	L	40%	20%
25	T	0.62	1.71	H	50%	10%

**Note:** NT = non-tumor; T = tumor; H = >10,000 cells; L = >5,000 and <10,000 cells.

**Figure 2:**
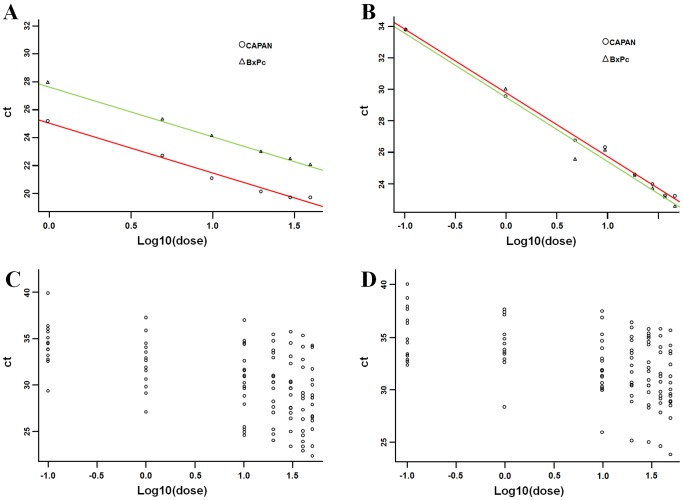
Identification of the minimal RNA quantity to obtain an adequate qRT-PCR reaction from cytology specimens. (**A**) The relationship between Ct and ldose for the two cell lines (BxPc3 and Capan-1) was analyzed as covariance (ANCOVA) model and the minimal RNA quantity was identified in terms of the estimated slope. For RNU6B, the ANCOVA analysis (R2 = 0.99) revealed that the slope of the relationship between ldose and Ct is the same in the two cells lines (p = 0.55) and it is equal to −3.73 (±0.07, p<0.0001), with efficiency *e* = 1.70. The estimated models are Ct = 27.65–3.55*ldose for BxPc3 and Ct = 24.97–3.55*ldose for Capan-1. (**B**) For miR-205 ANCOVA analysis (R2 = 0.98) revealed that the slope of the relationship between ldose and Ct is the same in the two cell lines and is equal to −4.00 (±0.12, p<0.000), with efficiency *e* = 1.50. The estimated model is Ct = 29.09–3.64*ldose both for BxPc3 and Capan-1. (**C**) To estimate the relationship between Ct and ldose on the 15 non-neoplastic patients, we used linear mixed effect models including a random effect for the subject. (**D**) The minimal RNA quantity was selected in terms of the fixed effect slope. For miR-205, when considering all the dilutions, the LME analysis (R2 = 0.92) revealed that the fixed effect slope of the relationship between ldose and Ct is −1.73 (±0.13, p<0.000), with efficiency *e* = 4.78.

To estimate the efficiency *e* of the qRT-PCR obtained by cytology smear and to test the relationship between Ct and ldose, a covariance analysis (ANCOVA) model was applied to the two cell lines (Capan-1 and BxPC3). The best cut-off for RNA amount to obtain a proper qRT-PCR analysis was selected in terms of the estimated slope (Table 2). All the diagnostics supported the estimated ANCOVA models. For RNA U6B, when considering all the tested dilutions (i.e., 50 ng, 40 ng, 30 ng, 20 ng, 10 ng, 5 ng, 1 ng, 0.1 ng), the ANCOVA analysis (R2 = 0.99) revealed that the slope of the relationship between ldose and Ct was the same in the two cell lines (p = 0.55) and it was equal to −3.73 (±0.07, p<0.0001), with efficiency *e* = 1.70. The Ct measurements were systematically lower in Capan-1 compared with BxPC3 (-2.68±0.12, p<0.0001). When considering amount of total RNA ≥1 ng, the ANCOVA analysis (R2 = 0.99) showed that the slope of the relationship between ldose and Ct was equal to −3.561 (±0.11, p<.0001), with efficiency *e* = 1.81. The estimated models are Ct = 27.65–3.55*ldose for BxPc3 and Ct = 24.97–3.55*ldose for Capan-1 (Figure 2A). For miR-205, when considering all the dilutions, ANCOVA analysis (R2 = 0.98) revealed that the slope of the relationship between ldose and Ct was the same in the two cell lines and was equal to −4.00 (±0.12, p<.0001), with efficiency *e* = 1.50. When considering amount of total RNA ≥5 ng, the ANCOVA model (R2 = 0.91) showed that the slope of the relationship between ldose and Ct was −3.649 (±0.39, p<.000), with efficiency *e* = 1.75. The estimated model was Ct = 29.09–3.64*ldose both for BxPC3 and Capan-1 (Figure 2B).

**Table 2:** RNA amount cut-off values for qRT-PCR analysis using archival cytology smears.

Material	Gene	RNA amount cut-off	Slope	R-squared	Efficiency
Cell lines	RNA U6B	1 ng	−3.561	0.99	1.81
	miR-205	5 ng	−3.649	0.91	1.75
Non-tumor urine smears	RNA U6B	10 ng	−2.966	0.99	2.37
	miR-205	20 ng	−3.408	0.99	1.94

### RNA Extracted from Cytology Smear is Suitable for qRT-PCR Analysis

To validate these results in long-stored urine archival smears, a total of 15 non-neoplastic urine smears were investigated, by applying a LME model including a random effect for the subject. The minimal RNA amount to perform an adequate qRT-PCR analysis was selected in terms of the fixed effect slope (Table 2). All the diagnostics on the residuals supported the estimated LME models. For RNA U6B, when considering all the tested dilutions, LME analysis (R2 = 0.92) revealed that the fixed effect slope of the relationship between ldose and Ct was equal to −2.43 (±0.29, p<0.000), with efficiency *e* = 3.11. When considering RNA amount ≥10 ng, the LME analysis (R2 = 0.99) showed that the fixed effect slope of the relationship between ldose and Ct was equal to −2.966 (±0.33, p<0.000), with efficiency *e* = 2.37. The estimated model was Ct = 33.23–2.966*ldose. For miR-205, when considering all dilutions, the LME analysis (R2 = 0.54) revealed that the fixed effect slope of the relationship between ldose and Ct was −1.73 (±0.13, p<0.000), with efficiency *e* = 4.78. When considering RNA amount ≥20 ng, the LME analysis (R2 = 0.99) showed that the fixed effect slope of the relationship between ldose and Ct was −3.41 (±0.36, p<.000), with efficiency *e* = 1.94. These data pinpoint 20 ng of total RNA as the minimum cut-off to use in miRNA expression profiling in archival urine cytology specimens.

### Archival Urine Cytology Smears for miRNA Expression Profiling Studies

**Figure 3:**
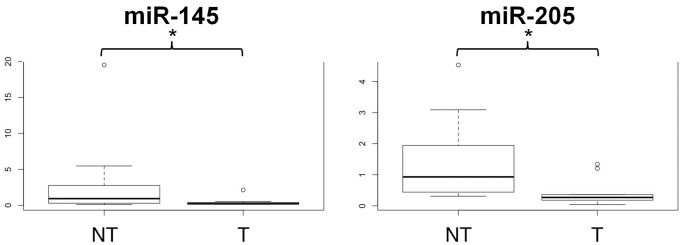
Total RNA extracted from archival urine cytology smears is suitable for miRNA expression profiling. Box plots show differences in miRNAs expression between non-tumor (NT) and low grade urothelial carcinoma samples (T). *p<0.05.

Both miR-145 and miR-205 were significantly down-regulated in UC specimens in comparison with non-neoplastic smears (−7.25 and −3.46, respectively; p<0.05) (Figure 3). These findings validate miRNA expression profiling by using total RNA extracted from archival urine specimens.

## Discussion

Formalin fixed and paraffin embedded (FFPE) tissues contain virtually all the information that confirm the central position of clinical pathology in diagnosis, prognosis, therapy recommendation, and disease mechanism insight. Well along in this pathway, cytopathology runs a parallel and successful track: many patients cannot undergo an open biopsy and cytological smears are the only tissue available for diagnosis. Moreover, disease and treatment modifications can be monitored via repeated sampling, as *e.g.* for urine in UC or effusions for carcinomas metastatic to serosal cavities.

UC involves the urinary tract and represents the fourth most common cancer in men in the western world. UC is characterized by frequent recurrences comprised between 50% and 70%, and 10–15% of patients develops muscle invasion over a 5-yr period, requiring in many cases a longtime surveillance [1].

The gold standard for primary detection and follow-up of UC consists of cystoscopy and cytology [31]. Cystoscopy identifies most of papillary and solid lesions, but it is invasive and uncomfortable for patients and causes a urinary tract infection in around 10% of the patients [32]. Urine cytology has a specificity of 93% to detect high-grade tumors with a reasonable sensitivity, but such sensitivity drops down to 4–31% for low-grade UC cases [7]. Therefore there is a clear need for more accurate urinary UC biomarkers.

As aforementioned, various molecular urinary biomarkers have been developed to improve UC detection; however, none of these showed a satisfying sensitivity or specificity for replacing cystoscopy, even coupled with cytology. A relatively new class of biomarkers (i.e. miRNAs) has been proposed for diagnostics, treatment monitoring and therapeutic approach in oncology.

In this paper, we first defined the minimal total RNA concentration for a reliable and reproducible miRNAs expression profiling analysis. Then, in order to demonstrate that archival cytology specimens are still informative for diagnostic purpose, we examined total RNA from 15 non-neoplastic and 10 UC cytology samples obtaining from each a good quantity (ranged between 0.06–4.44 µg) and good purity (OD_260/280 nm_ ratio between 1.7 and 2.3) of total RNA.

To further support the data, we analyzed the expression of miR-145 and miR-205, which have been recently associated to UC [12], [21]–[25]. Indeed, it has been reported that miR-145 expression is significantly reduced in resected specimens of low-grade, *in situ,* and muscle invasive UC [22], [25], while miR-205 is consistently down-regulated in progressing UC since its direct involvement in early bladder carcinogenesis [23], [24].

Our preliminary results noticeably reproduced in the archival urine specimens the findings obtained in resected samples corroborating the hypothesis that miRNAs expression profiling analysis is feasible and reliable even in such scant and degraded material.

Numerous, recent studies have explored circulating cell-free (mainly exosome-based) miRNAs and provided evidence that miRNAs exist in a stable form in various body fluids, such as blood, urine, saliva, and peritoneal fluid [15]–[18], [33]. Moreover, such papers have shown that miRNA signatures in body fluids are not affected by age and sex differences ruling out a possible bias in this study introduced by random selection of consecutive tumors and control urine cytology samples instead of specimens from matched populations [15], [16], [34]. Of interest, a recent study identified members of miR-200 family, miR-155, miR-192 and miR-205, as reliable UC biomarkers in fresh urine for long-term surveillance of UC patients [34]. It has to be underlined that, contrarily to fresh urine, cytology urine samples are always steadily available as they are stored in pathology and cytopathology archives for many years. Other intriguing miRNAs to be tested in large series of UC cases are miR-129, since its over-expression has been implicated in apoptosis avoidance by direct targeting of *SOX4* and *GALNT1* [22], and miR-200 family, since its down-regulation has been involved in the complex process known as epithelial-mesenchymal transition (EMT) [23], [26], [28].

Of interest, the method here presented, after a preliminary assessment of specimen cellularity and tumor cell ratio, could be virtually applied to whatever sample stored in cytopathology archives, from fine-needle aspirates to effusions and bronchoalveolar lavages. Further studies should evaluate this fascinating possibility.

### Conclusions

Our preliminary data opens a broad field of immediately available and low cost diagnostic samples to shed new light on urinary tract cancer. Larger studies should assess the wide spectrum of significant miRNAs involved in bladder cancer to be applied in clinical practice as diagnostic, prognostic, and predictive markers.

## References

[1] Witjes JA, Hendricksen K (2008) Intravesical pharmacotherapy for non-muscle-invasive bladder cancer: a critical analysis of currently available drugs, treatment schedules, and long-term results. Eur Urol 53: 45–52. 17719169 10.1016/j.eururo.2007.08.015

[2] Eble JN. (2004) Pathology and genetics of tumours of the urinary system and male genital organs. Lyon: IARC press.

[3] Fassan M, Trabulsi EJ, Gomella LG, Baffa R (2007) Targeted therapies in the management of metastatic bladder cancer. Biologics Dec 1: 393–406. PMC2721287 19707309

[4] Bastacky S, Ibrahim S, Wilczynski SP, Murphy WM (1999) The accuracy of urinary cytology in daily practice. Cancer 87: 118–128. 10385442 10.1002/(sici)1097-0142(19990625)87:3<118::aid-cncr4>3.0.co;2-n

[5] Brown FM (2000) Urine cytology. It is still the gold standard for screening? Urol Clin North Am 27: 25–37. 10696242 10.1016/s0094-0143(05)70231-7

[6] Koss LG, Deitch D, Ramanathan R, Sherman AB (1985) Diagnostic value of cytology of voided urine. Acta Cytol 29: 810–816. 3863429

[7] Tilki D, Burger M, Dalbagni G, Grossman HB, Hakenberg OW, et al (2011) Urine markers for detection and surveillance of non-muscle-invasive bladder cancer. Eur Urol 60: 484–492. 21684071 10.1016/j.eururo.2011.05.053

[8] Gutierrez Banos JL, del Henar Rebollo Rodrigo M, Antolin Juarez FM, Garcia BM (2001) Usefulness of the BTA STAT Test for the diagnosis of bladder cancer. Urology 57: 685–689. 11306381 10.1016/s0090-4295(00)01090-6

[9] Hwang EC, Choi HS, Jung SI, Kwon DD, Park K, et al (2001) Use of the NMP22 BladderChek test in the diagnosis and follow-up of urothelial cancer: a cross-sectional study. Urology 77: 154–159. 10.1016/j.urology.2010.04.059 20739046

[10] Daniely M, Rona R, Kaplan T, Olsfanger S, Elboim L, et al (2007) Combined morphologic and fluorescence in situ hybridization analysis of voided urine samples for the detection and follow-up of bladder cancer in patients with benign urine cytology. Cancer 111: 517–524. 17963263 10.1002/cncr.23119

[11] Calin GA, Croce CM (2006) MicroRNA signatures in human cancers. Nat Rev Cancer 6: 857–866. 17060945 10.1038/nrc1997

[12] Gottardo F, Liu CG, Ferracin M, Calin GA, Fassan M, et al (2007) Micro-RNA profiling in kidney and bladder cancers. Urol Oncol 25: 387–392. 17826655 10.1016/j.urolonc.2007.01.019

[13] Fassina A, Cappellesso R, Simonato F, Lanza C, Marzari A, et al (2012) Fine needle aspiration of non-small cell lung cancer: current state and future perspective. Cytopathology 23: 213–219. 22805511 10.1111/j.1365-2303.2012.01005.x

[14] Bartel DP (2009) MicroRNAs: target recognition and regulatory functions. Cell 136: 215–233. 19167326 10.1016/j.cell.2009.01.002 PMC3794896

[15] Yamada Y, Enokida H, Kojima S, Kawakami K, Chiyomaru T, et al (2011) MiR-96 and miR-183 detection in urine serve as potential tumor markers of urothelial carcinoma: correlation with stage and grade, and comparison with urinary cytology. Cancer Sci 102: 522–529. 21166959 10.1111/j.1349-7006.2010.01816.x

[16] Zhou H, Guo JM, Lou YR, Zhang XJ, Zhong FD, et al (2010) Detection of circulating tumor cells in peripheral blood from patients with gastric cancer using microRNA as a marker. J Mol Med 88: 709–717. 20349219 10.1007/s00109-010-0617-2

[17] Gilad S, Meiri E, Yogev Y, Benjamin S, Lebanony D, et al (2008) Serum microRNAs are promising novel biomarkers. PLoS One 3: e3148. 18773077 10.1371/journal.pone.0003148 PMC2519789

[18] Schaefer JS, Montufar-Solis D, Vigneswaran N, Klein JR (2011) Selective upregulation of microRNA expression in peripheral blood leukocytes in IL-10−/− mice precedes expression in the colon. J Immunol 187: 5834–5841. 22043014 10.4049/jimmunol.1100922 PMC3221883

[19] Fassina A, Cappellesso R, Fassan M (2011) Classification of non-small cell lung carcinoma in transthoracic needle specimens using microRNA expression profiling. Chest 140: 1305–1311. 21622546 10.1378/chest.11-0708

[20] Fassina A, Marino F, Siri M, Zambello R, Ventura L, et al (2012) The miR-17–92 microRNA cluster: a novel diagnostic tool in large B-cell malignancies. Lab Invest 92: 1574–1582. 22964854 10.1038/labinvest.2012.129

[21] Catto JW, Alcaraz A, Bjartell AS, De Vere White R, Evans CP, et al (2011) MicroRNA in prostate, bladder, and kidney cancer: a systematic review. Eur Urol 59: 671–681. 21296484 10.1016/j.eururo.2011.01.044

[22] Dyrskjot L, Ostenfeld MS, Bramsen JB, Silahtaroglu AN, Lamy P, et al (2009) Genomic profiling of microRNAs in bladder cancer: miR-129 is associated with poor outcome and promotes cell death in vitro. Cancer Res 69: 4851–4860. 19487295 10.1158/0008-5472.CAN-08-4043

[23] Kenney PA, Wszolek MF, Rieger-Christ KM, Neto BS, Gould JJ, et al (2011) Novel ZEB1 expression in bladder tumorigenesis. BJU Int 107: 656–663. 20735391 10.1111/j.1464-410X.2010.09489.x

[24] Neely LA, Rieger-Christ KM, Neto BS, Eroshkin A, Garver J, et al (2010) A microRNA expression ratio defining the invasive phenotype in bladder tumors. Urol Oncol 28: 39–48. 18799331 10.1016/j.urolonc.2008.06.006

[25] Ostenfeld MS, Bramsen JB, Lamy P, Villadsen SB, Fristrup N, et al (2010) miR-145 induces caspase-dependent and -independent cell death in urothelial cancer cell lines with targeting of an expression signature present in Ta bladder tumors. Oncogene 29: 1073–1084. 19915607 10.1038/onc.2009.395

[26] Wiklund ED, Bramsen JB, Hulf T, Dyrskjot L, Ramanathan R, et al (2011) Coordinated epigenetic repression of the miR-200 family and miR-205 in invasive bladder cancer. Int J Cancer 128: 1327–1334. 20473948 10.1002/ijc.25461

[27] Gray W, Kocjan G. (2010) Diagnostic cytopathology. China: Churchill Livingstone Elsevier.

[28] Fassina A, Cappellesso R, Guzzardo V, Dalla Via L, Piccolo S, et al (2012) Epithelial-mesenchymal transition in malignant mesothelioma. Mod Pathol 25: 86–99. 21983934 10.1038/modpathol.2011.144

[29] Rasmussen R. (2001) Quantification on the LightCycler. In: Meuer S, Wittwer C, Nakagawara K, editors. Rapid Cycle Real-time PCR, Methods and Applications. Heidelberg: Springer Press. 21–34.

[30] Magee L (1990) R2 measures based on Wald and likelihood ratio joint significance tests. Amer Stat. 44: 250–253.

[31] Babjuk M, Oosterlinck W, Sylvester R, Kaasinen E, Bohle A, et al (2011) EAU guidelines on non-muscle-invasive urothelial carcinoma of the bladder, the 2011 update. Eur Urol 59: 997–1008. 21458150 10.1016/j.eururo.2011.03.017

[32] Almallah YZ, Rennie CD, Stone J, Lancashire MJ (2000) Urinary tract infection and patient satisfaction after flexible cystoscopy and urodynamic evaluation. Urology 56: 37–39. 10869618 10.1016/s0090-4295(00)00555-0

[33] Valadi H, Ekstrom K, Bossios A, Sjostrand M, Lee JJ, et al (2007) Exosome-mediated transfer of mRNAs and microRNAs is a novel mechanism of genetic exchange between cells. Nat Cell Biol 9: 654–659. 17486113 10.1038/ncb1596

[34] Wang G, Chan ES, Kwan BC, Li PK, Yip SK, et al (2012) Expression of microRNAs in the urine of patients with bladder cancer. Clin Genitourin Cancer 10: 106–113. 22386240 10.1016/j.clgc.2012.01.001

